# Quality evaluation of health information about breast cancer treatment found on WeChat public accounts

**DOI:** 10.1186/s13690-023-01184-2

**Published:** 2023-09-15

**Authors:** Wenjuan Yang, Bingyan Li, Min Liu, Dongtong Tong, Yang Zou, Xing Li, Lunfang Xie

**Affiliations:** 1 School of Nursing, Anhui Medical University, Hefei, China; 2 The First Affiliated Hospital of Anhui Medical University, Hefei, China

**Keywords:** Breast cancer, Treatment health information, Quality evaluation, WeChat public accounts

## Abstract

**Background:**

With growing cases of breast cancer, WeChat public account, an important information publishing platform of WeChat, has become a breast cancer treatment health information provider to a huge audience. It is essential for health information to possess high-level accuracy and reliability. This work evaluates the quality of health information on breast cancer treatment in WeChat public accounts (WPAs), to benefit the patients while making treatment decisions and provide WPA authors with suggestions on publishing high-quality treatment health information.

**Methods:**

With “breast cancer” as keywords, searches were implemented on weixin.sogou.com and the WeChat app. The WPAs oriented to patients with breast cancer were selected, and the four latest articles of each WPA were included in a set to be evaluated with DISCERN.

**Results:**

A total of 37 WPAs and 136 articles published by them were included. The accounts operated by individual users were 54%. The median of overall quality of 136 articles was 44 (interquartile range = 10.75) and ranked as “fair”, of which only 28 (21%) were of “good” or higher quality. Among these articles, 74 (54%) were related to medical treatments, and 13 of them mentioned clinical trials; 36 (27%) dealt with surgery. 101 (74.26%) omitted additional sources of information; 102 (75%) did not explicitly suggest shared decision-making. A significant difference was not found in the dimensions “reliability of the articles” and “specific details of information on treatment choices” between the distinct categories of account subjects and various treatment options (*P* > 0.05).

**Conclusions:**

The quality of the articles on breast cancer treatment health information in WPAs was moderate. WPA producers should focus on improving the reliability of information and providing more details on treatment options, to assist patients in making optimal decisions during treatment.

**Supplementary Information:**

The online version contains supplementary material available at 10.1186/s13690-023-01184-2.

**Table:** 

**Text box 1. Contributions to the literature**
• The quality of breast cancer treatment information on WeChat public accounts is moderate.
• Most breast cancer treatment information does not support shared decision-making.
• The majority of breast cancer treatment information does not provide additional sources.
• Authors should clarify the advantages and disadvantages of various surgical options.

## Background

Breast cancer (BC) is the most common malignant tumor affecting women worldwide. According to GLOBOCAN statistics, there were approximately 2.3 million new cases worldwide in 2020, accounting for 11.7% of all cancer cases [1]. In China, BC is also the most common malignancy in women, with an incidence rate of 59.0/100,000 in 2020 [2]. The 5-year survival rate of Chinese patients with BC increased by 7.3% in the past 10 years to 83.2% [3]. However, there is treatment delay to varying degrees among Chinese patients with BC, owing to insufficient disease awareness [4]. If patients are provided with accurate, understandable, and individualized treatment health information, they are not only more cooperative with treatment but also more actively involved in those decisions [5]. Furthermore, studies have shown that patients’ active participation in making treatment decisions improves both satisfaction with the decision-making processes and perceptions of quality of life after treatment [6–9]. Patients with a certain disease will have a long-term and stable demand for information, and additionally, physicians will encourage them to search for health information related to their own medical conditions [10].

The rapid development of the Internet and social media has dramatically changed the way the public access health information. In China, 76.3% of the public use computers to access health information on online websites [11]. Meanwhile, social media is becoming more and more popular to obtain health information. WeChat is the most popular mobile social media platform in China with 1.09 billion daily users (up to January 2021) [11, 12]. As a module of WeChat, WeChat public accounts (WPAs) gained 360 million readers and over 20 million registered accounts [12]. WPA has become an indispensable information dissemination platform for Chinese government agencies, medical institutions, enterprises and individuals [13]. A large amount of health information generated on WPAs has immense potential to affect the public’s health. A study showed that WeChat was a frequent health information source for approximately 1/3 of the population and the most desirable access to health information for 63.26% of respondents [11]. In China, 44.02% of patients with BC obtain online health information in the preliminary stages of treatment, and 66.7% of them search the WeChat platform [11]. Furthermore, patients with BC pay long-term attention to related WPAs [14].

BC treatment options depend on comprehensive consideration of the tumor stage, the patient’s physiological conditions and underlying diseases, the patient’s willingness, and adverse reactions [15, 16]. The treatment options include surgery, medical, and complementary and alternative medicine (CAM) [17, 18]. Mastectomy and breast conservation surgery with radiotherapy are the most common treatments [17]. A systematic review revealed that patients with BC might regret treatment decisions after breast reconstruction due to the inferior quality of health information received preoperatively [19]. The higher the quality of health information, the more patients benefit, and to a deeper extent. Conversely, inaccurate, or incomplete health information on the Internet has a negative impact on the treatment decision-making of patients with BC; therefore, patients are skeptical about the quality of online health information after treatment [5, 20–22].

Studies have been conducted on the quality evaluation of health information about BC treatment, including patients’ surgical decisions [23], breast reconstruction post mastectomy [24], BC treatment options [17], and adverse effects of BC treatments [22]. However, these studies only concentrated on the quality of information selected from website, and there was a report that only 44.9% of Chinese patients with BC were satisfied with online health information [25–27].

Different from treatment information, health information covers a wider range of content, and the proportion of classifications of article providers in WPA is also different from that in websites. As WPAs have a considerable influence on the public, in this work, we rated BC treatment articles from WPAs with DISCERN, to evaluate their overall quality, find their merits and demerits, and accordingly, provide references for improving the quality of health information on BC treatment.

## Methods

### WPAs and article collection

We first retrieved articles and WPAs with the keywords “breast cancer” from both Sogou WeChat website (https://weixin.sogou.com/) and WeChat client and obtained WPAs by tracing the publisher of articles. In this study, WPAs related to BC were collected on April 11, 2021. We included the WPAs those WeChat profile page clearly indicated that they provided health information for patients with BC. The exclusion criteria were as follows: the WPAs (1) were duplicate; (2) had not updated for more than one year; (3) delivered only academic research information to professionals; (4) whose target population were not only patients with BC. Of 1332 retrieved WPAs, 41 were included in the analysis.

**Fig. 1:**
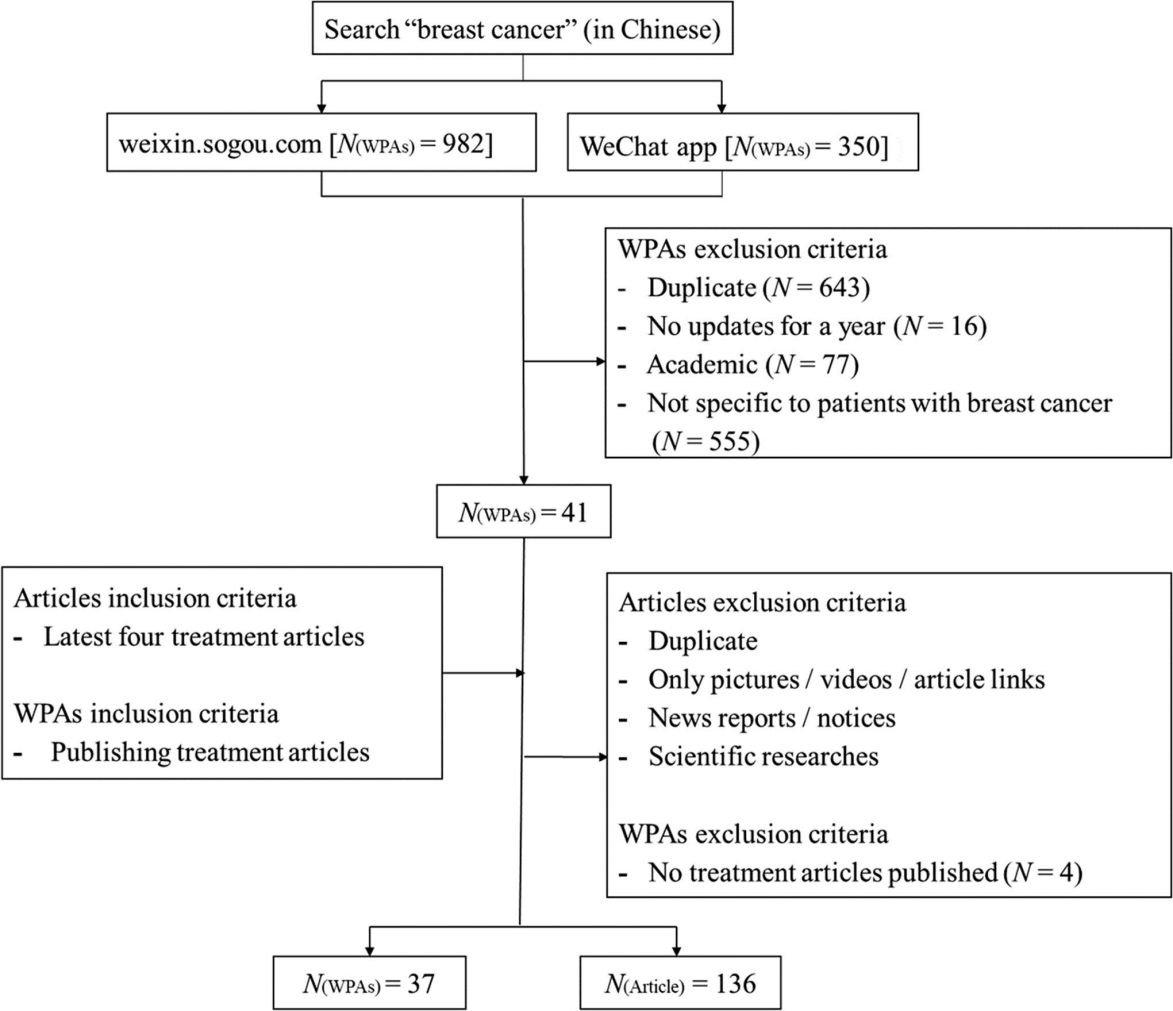
Processes of screening WeChat public accounts (WPAs) and treatment articles

Articles on BC treatment were selected from the 41 WPAs. To avoid bias, up to four newly published articles were included from WPAs as required below: articles on health information for BC treatment, and excluding (1) duplicate articles; (2) publications with only pictures, videos, or links; (3) news reports or notices; (4) academic articles. Finally, 136 articles published by 37 WPAs were included; four WPAs were further excluded because they lacked BC treatment articles. Figure 1 showed the screening processes for WPAs and treatment articles.

### Evaluation tools

#### Basic characteristics of information table

The following information was collected: WPA names, account subjects, article titles, and treatment options. Account subjects, as listed on the WPAs’ profile page, were categorized into individuals (e.g., physicians), enterprises (e.g., companies), institutions (e.g., public hospitals), and non-profit organizations (e.g., academic conferences). BC treatment options were classified as surgery, medical, and CAM. An additional mark was needed to determine whether a clinical trial was involved when a treatment option was classified as “medical”. Two researchers collected the information from every selected article and reached agreements through discussion.

#### DISCERN

DISCERN arose from a national project, and was developed by Charnock et al. [28] in 1999 to establish quality thresholds for treatment information. It summarizes quality indexes from general characteristics of treatment publications, and was widely applied to online information. It consists of three parts and 16 items. The first part (Items 1–8) is related to the reliability of the article; the second part (Items 9–15) evaluates the specific details of treatment choice information; the third part (Item 16) describes the overall quality of the source publication of health information on treatment choices. Items are scored based on a 5-point Likert scale (see Ref. [23] for detailed criteria): good (4–5 points), fair (3 points), and poor (1–2 points) [29]. The quality of an article is ranked into 5 grades based on positively correlated DISCERN total points (16–80 points): very poor (≤ 29 points), poor (30–40 points), fair (41–51 points), good (52–63 points), and excellent (64–80 points) [30, 31]. Two raters individually assessed the quality of each article. Cohen’s kappa statistic was used to evaluate the inter-rater reliability. Of the 136 articles evaluated by DISCREN, Cohen’s kappa coefficient was 0.78, and ranged from 0.62 to 0.93 for each item; the consistency strength was ranked as “substantial” [32].

### Rating process

To assess the operability of the evaluation, Raters 1 (WY) and 2 (BL) assessed five identical articles referencing the DISCERN Handbook, which were randomly selected from the included articles. The raters improved their understanding of scoring criteria through discussion on the controversial item scores. To simulate the situations in which patients read WPA articles and ensure the consistency and integrity of assessment, the researchers sent the selected treatment articles to a WeChat group, read the articles on the WeChat app, and implemented quality evaluations. If there was an inconsistency in the scores of an item, the two raters would reach a consensus; otherwise, the research group reached a final decision through discussion.

### Statistical analysis

Statistical analyses were performed with IBM SPSS Statistics 24.0 and Microsoft Office Excel 2016. The quality scores of 136 articles were described using median and interquartile range (IQR). The statistics were described using frequency, constituent ratio, and rate. Rank-sum test was used to compare the scores of article qualities and DISCERN items between the diverse categories of account subjects and different treatment options. *P*-value < 0.05 indicated statistical significant difference.

## Results

### Characteristics of included WPAs and articles

In the aspect of account subject, the 37 WPAs were classified into four categories: individual users, accounting for the largest proportion (20, 54%), of which 14 were operated by clinicians; enterprise (8, 22%); institution (6, 16%); non-profit organization (3, 8%). The number of individual users’ publications also occupied a dominant position compared with the other three categories. An additional file showed this in more detail [see Additional file 1]. Among these BC treatment articles, the most focused treatment was medical, with 74 articles (54%), of which 3 introduced the clinical guidelines on BC, and 13 involved clinical trials. Moreover, 36 articles (27%) referred to surgical treatments, and 16 articles (12%) introduced more than one option, mostly surgical and medical treatments.

### Quality analyses

**Fig. 2:**
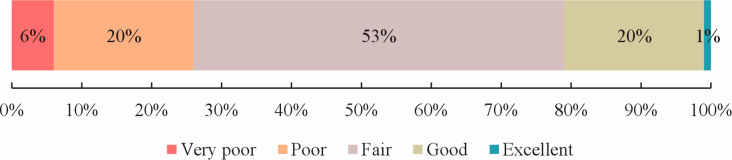
Distribution of grades for DISCERN scores of articles (n = 136)

The total DISCERN scores of 136 articles range from 21 to 65 with a median of 44 (IQR = 10.75), indicating that the quality of BC treatment health information was “fair”, of which only 28 (21%) were of “good” or higher quality. The quality distribution of the articles was shown in Fig. 2. Table 1 listed the grade distribution and median (and IQR) of each DISCERN item of the included articles. Only four items were of high quality (median ≥ 4). In the DISCERN tool, the evaluation of treatment health information consists of two dimensions: reliability of the article (Part 1) and specific details of information on treatment choices (Part 2). In the reliability dimension, the item with the worst quality was “provides additional sources of information” (*n* = 101, 74.26%); the general median score of these articles was 3.00 (IQR = 0.59), ranked as “fair”. In the detail dimension, the item with the worst score was “supports shared decision-making” (*n* = 102, 75%), and the general median score was 2.57 (IQR = 1), graded between “poor” and “fair”.

**Table 1:** Evaluation results of each item in DISCERN

DISCERN item	Distribution of score level, *n* (%) ^a^			Median (IQR ^b^)
	Poor	Fair	Good	
**Part 1: reliability of the article**				
1. Provides clear aims	34 (25.00)	48 (35.29)	54 (39.71)	3 (1.75)
2. Achieves its aims ^**c**^	4 (3.39)	14 (11.86)	100 (84.75)	4.5 (1)
3. Provides relevant information	10 (7.35)	29 (21.32)	97 (71.33)	4 (2)
4. Provides sources of information	99 (72.79)	19 (13.97)	18 (13.24)	2 (2)
5. Provides information production date	88 (64.71)	40 (29.41)	8 (5.88)	2 (1)
6. Is balanced and unbiased	16 (11.76)	90 (66.18)	30 (22.06)	3 (0)
7. Provides additional sources of information	101 (74.26)	0 (0)	35 (25.74)	2 (2)
8. Refers to areas of uncertainty	81 (59.56)	34 (25.00)	21 (15.44)	2 (1)
**Part 2: specific details of information on treatment choices**				
9. Describes how each treatment works	40 (29.41)	19 (13.97)	77 (56.62)	4 (3)
10. Describes benefits of each treatment	41 (30.15)	25 (18.38)	70 (51.47)	4 (3)
11. Describes risks of each treatment	67 (49.26)	21 (15.44)	48 (35.30)	3 (3)
12. Describes what would happen if any treatment is not used	90 (66.18)	16 (11.76)	30 (22.06)	1 (2)
13. Describes how treatment affects quality of life	89 (65.44)	11 (8.09)	36 (26.47)	1 (3)
14. Clarifies that there may be more than one treatment choice	41 (30.15)	43 (31.62)	52 (38.23)	3 (2.75)
15. Supports shared decision-making	102 (75.00)	12 (8.82)	22 (16.18)	1 (1.75)
**Part 3:**				
16. Overall quality	38 (27.94)	80 (58.82)	18 (13.24)	3 (1)

^**a**^ Rank based on a 5-point Likert scale: good (4–5), fair (3), and poor (1–2)

^**b**^ IQR: interquartile range

^**c**^ Excluding 18 articles without clear aims

### Comparisons within distinctive characteristics

Table 2 presents quality level comparison of account subjects and treatment options. The proportion of articles ranked as “fair” and above levels was the highest for institution accounts, 20 in total 24, while it was the lowest for enterprise accounts (18 in 29). In the categories classified based on treatment options, the proportion of “fair” and above articles was the highest (6 in 7) in the category of surgical + medical + CAM treatments, and it was the lowest (5 in 9) in the category of surgical + medical treatments. Statistical significant differences were not found in the quality levels either among account subject categories or among treatment options, and thus there was no need to use Bonferroni correction for multiple comparisons to control family-wise error rate. Meanwhile, the quality of surgical treatment articles was ranked as “fair”; 3 medical treatment articles related to clinical guidelines had “poor” or “fair” grades; clinical trial articles had a “very poor” grade with a median score of 29 (IQR = 12). As for DISCERN-part score comparison (Table 3), both the reliability and specific details of treatment choices information of all account subjects or treatment options were at the levels of “fair” and below. Statistical significant differences were not found in the DISCERN-part scores either among account subject categories or among treatment options, so that Bonferroni correction was unnecessary.

**Table 2:** Quality levels of articles and comparison of quality levels of account subjects and treatment options

Category	Sum score, Median (IQR ^b^)	Quality level ^a^					*H*	*P*
		Very poor	Poor	Fair	Good	Excellent		
**Account subject of WPAs**							1.855	0.603
Individual	44.00 (9.00)	1	16	42	12	-		
Enterprise	44.00 (15.00)	4	7	12	5	1		
Institution	44.50 (11.50)	2	2	14	6	-		
Non-profit organization	44.50 (14.75)	1	3	4	4	-		
**Treatment option in articles**							6.244	0.182
Surgical	45.00 (9.75)	-	4	12	4	-		
Medical	44.00 (10.50)	8	14	36	15	1		
CAM ^**c**^	47.00 (9.25)	-	5	17	4	-		
Surgical + Medical	42.00 (10.00)	-	4	5	-	-		
Surgical + Medical + CAM	54.00 (13.00)	-	1	2	4	-		

^**a**^ Ranked by score: very poor (≤ 29), poor (30–40), fair (41–51), good (52–63), excellent (64–80)

^**b**^ IQR, interquartile range

^**c**^ CAM, complementary and alternative

**Table 3:** Comparison of DISCERN scores across parts by account subjects and treatment options

Category	DISCERN item mean score ^a^ [median (P25, P75)]	
	Reliability of articles (Items 1–8)	Specific details of treatment choices information (Items 9–15)
Account subject of WPAs		
Individual	2.88 (2.75, 3.25)	2.57 (2.29, 3.14)
Enterprise	3.13 (2.47, 3.44)	2.57 (1.86, 3.07)
Institution	3.13 (2.66, 3.25)	2.57 (2.18, 3.25)
Non-profit organization	3.07 (2.69, 3.47)	2.43 (1.90, 3.61)
*H*	1.078	0.886
*P*	0.782	0.829
**Treatment option in articles**		
Surgical	2.88 (2.66, 3.25)	2.57 (2.33, 3.14)
Medical	3.00 (2.60, 3.28)	2.50 (2.00, 3.14)
CAM ^**b**^	3.00 (2.72, 3.28)	2.71 (2.40, 3.14)
Surgical + medical	3.00 (2.59, 3.25)	2.14 (2.07, 2.50)
Surgical + medical + CAM	3.13 (2.75, 3.63)	3.14 (2.57, 3.86)
*H*	1.119	10.211
*P*	0.891	0.37

^**a**^ Rank based on a 5-point Likert scale: good (4–5), fair (3), and poor (1–2)

^**b**^ CAM, complementary and alternative medicine

## Discussion

In China, WPAs are the most popular health education channels with a high affinity and influence [11]. In the past, quality assessments of health information in WPAs mostly focused on accounts related to comprehensive health information (nonspecific diseases) and online HPV vaccine information [33, 34]. To the best of our knowledge, this is the first study to evaluate the quality of BC treatment health information in WPAs with DISCERN.

### Individual account: a large proportion of articles published

To ensure the authenticity and security of WPAs, Tencent (the developer of WeChat) provides account authentication services so that readers have access to the subject category of a WPA in its introduction. Account subjects were classified into individual, enterprise, institution, and non-profit organizations. Enterprise accounts refer to those that sell services for profit (e.g., companies providing health services), like commercial accounts shown in search engines, such as Google and Baidu [17, 25]. Institutions were represented by public hospitals and their relevant departments. Moreover, institutional accounts regularly send updates to subscribers and are important health information providers [13, 35].

In the quality evaluation of the information on Google regarding breast reconstruction after mastectomy [24], it was found that most websites included in the study were commercial. Studies on health information in search engines, performed by other Chinese scholars, further showed that business organizations accounted for the majority of the included account subjects and individuals were of low proportion [25, 36]. Nevertheless, in this study, individual users were of the highest proportion (54%) of the treatment option publications, among whom clinicians (breast surgeons, medical oncologists, etc.) were the majority. This may result from the low entry threshold for WPAs, where an individual applicant obtains a WPA registration after only submitting identity information (e.g., ID card number), mobile phone number, and bank card number linked to a WeChat ID. It encourages more individual publishers to disseminate additional health education information on WPAs, which is also verified in this study. In China, medical practitioners are the main providers of patient health education. By publishing articles in new media such as WeChat and Douyin (Chinese version of TikTok), they not only realize the systematic popularization of health science and expand its audience, but also improve the influence and effect of health communication. More importantly, their participation increases the rigor and scientificity of health science promotion [37]. With more medical practitioners devoted to new media platforms, in the future, health science promotion will increasingly develop with quality, precisely meet the demands of the public for health information and help to improve public health.

### Proportion of the treatment articles regarding on surgery

Currently, surgery is the most common treatment for BC. Most patients face a difficult choice between mastectomy and breast conservation surgery [23, 38]. However, only about 27% of the articles in this study discussed relevant information on surgery, and the quality grade was “fair”. It might be difficult for patients to obtain sufficient information on surgical treatment. A qualitative study of treatment decisions for patients with BC in China suggested that those affected are more likely to accept treatment decisions passively because of a lack of professional knowledge on cancer treatment [39]. For example, patients with BC undergoing mastectomy must decide whether and when to have breast reconstruction. However, patients undergoing breast reconstruction are generally not content with obtained information on potential postoperative complications, causing them to regret previous decisions after surgery [19]. Therefore, based on practical health education requirements of patients, WPA authors should concentrate on preparing high-quality health information that clarifies the advantages and disadvantages of various surgical options and helps the patients make treatment choices.

### Quality of BC treatment health information in WPAs

A DISCERN score ≥ 52 is a sign of a potential high-quality article. In this study, the median DISCERN score of WPA articles was 44 (IQR = 10.75), indicating that the quality of most BC treatment health information was at a medium level. This was similar to Dee et al.’s results of a health information quality assessment on the side effects of various BC treatments on Google [22]. Of the articles included in this study, items “achieves its aims” and “provides relevant information” scored the highest, proving that most articles have offered information on treatment options that readers expected from the aims of the articles, and these articles have addressed the problems focused on by most patients.

Specifically, this study found that WPAs lacked information reliability and sufficient details on treatment integrity. Most articles did not specify the sources of evidence for essential information, which indicated that the health information was still insufficient for detailed disclosure. As one of the important signs that reflect the objectivity of an article, the absence of evidence sources will directly interfere with patients’ judgment on the accuracy of the information, thereby affecting the dissemination and reception of health information [40]. Most articles did not provide additional information on treatment options which was problematic. We suggest that the authors should attach links titled “expand reading” or “useful address” at the end so that patients can learn more about available or potential treatments. A study has shown that 73% of patients with BC seek online information about alternatives and side effects of cancer treatments [41]. Better participation in treatment decisions can be achieved only when patients thoroughly comprehend all outcomes of available treatment options and compromise between their benefits and risks [23]. Patients’ initiative is encouraged when health information is patient-centered and non-biased [42].

For patients with BC, treatment decision-making is complex and highly dependent on their medical conditions, treatment processes, and personal preferences [43, 44]. When patients face multiple treatment options, shared decision-making emphasizes that patients should consult with surgeons, medical staff, and families to prevent them from making medical choices at will [44]. We discovered that most articles did not explicitly suggest that patients should discuss problems encountered during treatment with medical workers, families, or caregivers. These articles ignored the positive effects of shared decision-making on patients. Family makes an enormous difference in the emotional regulation of Chinese patients with BC and provides them with strong psychological support during treatment [39]. Furthermore, adequate emotional support increases patients’ confidence in their treatment options [45]. Moreover, this study found that most articles did not elaborate on the impact of treatment options on quality of life, which could hinder patients from better treatment decisions. Therefore, WPA authors should objectively describe the impacts of various treatment schemes on life and provide suggestions that patients should discuss all types of acquired information with medical staff or others to make the best decisions under self-management.

### Comparison of quality of articles published by different account subjects

This study further demonstrated that the proportion of articles with “fair” and above grades published by institutions was higher than that of enterprise accounts. This result was similar to the study on the quality assessment of Chinese health information on BC in Baidu, which showed that health information published by professional medical institutions had higher quality [25]. It was because the authors of institution accounts were professionals with rich medical knowledge and clinical experience. Their writing is based on sufficient evidence and experience and is unlikely to be affected by commercial interests, which improves the reliability and effectiveness of health information [25, 46]. Hence, medical staff should encourage patients to subscribe to the WPAs of professional medical organizations (e.g., top public hospitals) and read articles published by them. Enterprise accounts should enhance cooperation with professional medical organizations, such as inviting medical experts to review the contents and soliciting manuscripts from specialists, to ensure objective, authoritative, and up-to-date health information on BC treatment.

### Quality of articles about clinical guidelines and clinical trials

Clinical guidelines present standard treatment methods, so they should be introduced to the public in detail and in plain expressions. But in our study, there were only three articles involving clinical guidelines on breast cancer and the quality was fair or poor. The reason for this result was that these articles only displayed the updated treatment methods without specific details about treatment choices required by patients. We also noted that quality of articles about clinical trials was negative. Clinical trials are alternatives for patients with terminal cancer. A study showed that 55.9% of patients with cancer receive preliminary information about clinical trials from online articles or advertisements [47]. However, < 3% of Chinese patients with cancer have been involved in various clinical trials. In 2021, the Center for Drug Evaluation of NMPA (Chinese National Medical Products Administration), released a guideline that clinical trials should meet patients’ needs and be performed ethically [48]. In this study, only 13 articles (10%) referred to clinical trials, and they were all graded as “very poor.” These articles generally listed only project names, screening criteria of patients, and contact information of researchers, but lacked detailed project content. Moreover, medical terms frequently appeared in these articles and confused patients, which was detrimental to their decisions regarding treatment. Therefore, it is essential for WPA producers to accurately demonstrate the aims, methods, curative effect, potential unexpected reactions, and corresponding burden and efforts of the clinical trials, so that patients can understand the impact of these trials on their medical situation. Furthermore, producers should hire experts to revise the articles and evaluate their readability to publish reliable and understandable articles.

### Limitations

Restricted by the characteristics and functions of the WeChat platform, the search for WPAs pushing BC health information was not exhaustive. In addition, the selected articles could not reflect the well-rounded circumstances of BC health information on the WeChat platform. However, we intended for better representativeness via multiple accesses to WPAs that patients might browse, and we employed a standard scale to evaluate the quality of BC treatment articles. There might be other factors that have influence on the quality of information, such as effective interactive designs, individualized content, and attractive layouts, which could not be measured by DISCREN. These might be other dimensions for further research on article quality.

## Conclusions

This study indicates that the quality of BC treatment health information in WPAs is at a moderate level, and the reliability of articles and detailed information on treatment selection need to be further improved. To improve the quality of the articles, WPA producers should specify the sources of evidence for essential information, provide additional information about treatment options and the impacts of different treatment options on the quality of life, emphasize shared decision-making and its positive effects, publish more articles related to surgery, and pay special attention to the readability of the content of clinical trials.

### Electronic supplementary material

Below is the link to the electronic supplementary material.

Supplementary Material 1

## Data Availability

The datasets supporting the conclusions of this article are included within the article and its additional file.

## Abbreviations

BC: Breast cancer
WPAs: WeChat public accounts
CAM: Complementary and alternative medicine
