# Supplementary material for: Quality evaluation of health information about breast cancer treatment found on WeChat public accounts
Source: Arch Public Health. 2023 Sep 15;81:170. doi: 10.1186/s13690-023-01184-2 (PMC10503205; doi:10.1186/s13690-023-01184-2)
Supplement: Supplementary file 1 — Supplementary Material 1 [file 13690_2023_1184_MOESM1_ESM.docx]

| Article statistics of account subject categories and treatment options(n=136) | | | | | |
| --- | --- | --- | --- | --- | --- |
| **Treatment option** | **Account subject of WPAs** | | | | **Total** |
|  | **Individual** | **Enterprise** | **Institution** | **Non-profit organization** |  |
| Surgical | 13 | 3 | 3 | 1 | 20 (15%) |
| Medical | 34 | 17 | 16 | 7 | 74 (54%) |
| CAM **^a^** | 14 | 5 | 4 | 3 | 26 (19%) |
| Surgical + medical | 5 | 3 | 1 | 0 | 9 (7%) |
| Surgical + medical+ CAM | 5 | 1 | 0 | 1 | 7 (5%) |
| Total | 71 (52%) | 29 (21%) | 24 (18%) | 12 (9%) | 136 (100%) |
| **^a^** CAM, complementary and alternative medicine | | | | | |
